# Decomposing Differences of Health Service Utilization among Chinese Rural Migrant Workers with New Cooperative Medical Scheme: A Comparative Study

**DOI:** 10.3390/ijerph18179291

**Published:** 2021-09-02

**Authors:** Dan Li, Liang Zhu, Jian Zhang, Jinjuan Yang

**Affiliations:** 1 School of Public Management, Northwest University, Xi’an 710127, China; dandanli@nwu.edu.cn; 2 Department of Health Care Management and Medical Education, School of Military Preventive Medicine, Air Force Medical University, Xi’an 710032, China; 3 School of Public Policy and Administration, Xi’an Jiaotong University, Xi’an 710049, China; zhjian.2004@stu.xjtu.edu.cn; 4 School of Public Health, Health Science Center, Xi’an Jiaotong University, Xi’an 710061, China; yangjinjuan@stu.xjtu.edu.cn

**Keywords:** comparative analysis, health service utilization, Chinese rural migrant workers, new rural cooperative medical insurance

## Abstract

The New Rural Cooperative Medical Insurance (NCMS) in China has provided benefits for rural migrant workers’ health service utilization, but the financial coordination and mutual aid of NCMS is mainly based on the county or district as a unit, leading NCMS with the characteristics of regional segmentation. Our study aims to explore their health service utilization, as well as to decompose differences of the health service utilization into contributors. Data from the China Labor-Force Dynamic Survey in 2016 and Urban Statistical Yearbook in 2016 were used. We used coarsened exact matching to control the confounding factors in order to enhance the comparison of two groups. The Fairlie decomposition method was used to analyze the differences and the sources of health service utilization. Influencing factors of health service utilization for rural migrant workers with NCMS were diversified, especially contextual characteristic and individual characteristics. The proportion of ethnic minorities, the number of medical institutions for 10,000 people in the community, the number of beds for 10,000 people in the city, and the urban service quality index were the major contributors of the differences. The proportion of difference in the health service utilization of rural migrant workers with NCMS caused by health service need were −54.73% and 6.92%, respectively. The inequities of the probability of two weeks outpatient, and the probability of inpatients, were −0.006 and −0.007, respectively. There were substantial differences in the health service utilization between rural migrant workers with NCMS in the county/district and rural migrant workers with NCMS across the county/district. Our results illustrated the inequity from the differences on basis of characteristic effect and the discrimination effect. Our studies clarified that health service needs of should be fully considered, contributing to a more reliable understanding of the health service utilization of rural migrant workers.

## 1. Background

Chinese rural migrant workers have emerged since the implementation of new policies of reform and opening up to the outside world in China. With the continuous deepening of China’s industrialization and urbanization, the number of Chinese rural migrant workers is increasing. As a marginal group in China, Chinese rural migrant workers have the dual attributes of rural household registration (Hukou) and workers, and thus they usually face natural potential vulnerabilities such as great intensity of work, low salary, unstable work, poor living environment, and limited access to health service utilization.

The New Rural Cooperative Medical System (NCMS) is a basic medical insurance system for residents in rural China, designed to solve the problem of “suffering from poverty and returning to poverty by illness” in China. It is guided by the Chinese Government, and it adheres to the principle of voluntary participation. Individual, collective, and government co-financing compensates for the rural basic medical insurance system which mainly has the more serious medical expenses. NCMS gathers the power of the government, commercial insurance agencies, and medical institutions to share health risks of rural residents, so NCMS has the characteristic of mutual aid. To a certain extent, NCMS reduces the economic burden by illness of farmers and provides better access to health services.

NCMS in China has provided benefits for the health service utilization of rural residents. However, the financial coordination and mutual aid of NCMS is mainly based on the county or district as a unit, leading NCMS with the characteristics of strong regional segmentation. Rural migrant workers with NCMS without a local Hukou would have less access to health service utilization in destination county/district than those with a local Hukou. With the continuous deepening of local urbanization in the immediate vicinity, the number of rural migrant workers who choose to work in the immediate vicinity is increasing. Rural migrant workers with NCMS across the county/district utilized the health service outside the unified county/district, so the regional segmentation of NCMS would make a difference to the rural migrant workers across the county/district, not the rural workers in the county/district. Therefore, our attention should not be only the health service utilization of rural migrant workers, but also on the difference in health service utilization between rural migrant workers in the county/district and rural migrant workers across the county/district.

Previous studies have paid more attention on NCMS. Wagstaff et al. [1] used the method of “differences-in-differences” to compare the differences in health service utilization before and after the implementation of NCMS. They found NCMS promoted the probability of outpatient and the probability of inpatient, but it did not reduce expenses to rural patients. By a comprehensive assessment of NCMS, Brown et al. [2] believed that the basic characteristics of NCMS in different counties/districts were different, and the level of medical insurance compensation varies greatly between different counties/districts. Yu et al. [3] concluded that there was no significant correlation between NCMS and actual medical visits, they found that the rural residents with a good economic level had a higher hospitalization rate than the poor. The outline of “healthy China 2030” clearly proposed to reduce the difference of the utilization of basic public health services among different groups. However, there is still a lack of attention to the internal heterogeneity of rural migrant workers with NCMS.

Roemer et al. believed that reasonable inequalities should be allowed, and the compensation should be given for unequal treatment caused by factors beyond the control of individuals [4,5]. According to Fleurbaey et al. [6], the difference in health services is influenced by two factors: individual effort factors such as the need and preference for the health care, and environmental factors such as socio-economic status and social policy. Cook et al. [7] analyzed the differences in mental health services among White, Black, and Latino groups, and divided factors into three categories: needs and preferences; socioeconomic status and social environment; and discrimination between different ethnic groups. Consistent with Roemer, Fleurbaey, and Cook, we tried to study the equity of health services for rural migrant workers with NCMS. The statistical data cannot reflect the inequality in health services of rural migrant workers. Factors of reasonable factors and unreasonable factors should be accurately distinguished, that is to say, we should make the need of health service clear. Gu et al. [8] combined the “equal opportunity theory” with the Oaxaca-Blinder decomposition to study the difference of health service cost between urban residents and rural residents. Li et al. [9] used Fairlie decomposition to study health differences between older rural migrant workers and rural residents. There are some studies on the equity of access to health services between rural-to-urban migrant workers and household registration residents under the dual structure of urban and rural areas [9,10]. Unfortunately, little attention has been paid to the need of health service utilization in the sub-groups of rural migrant workers according to the “regional segmentation” of the NCMS. What’s worse, limited evidences have been conducted to control confounding factors between rural migrant workers in the county/district and rural migrant workers across the county/district by using CEM.

The Anderson model provided a good theoretical analysis framework for understanding the health service utilization [11,12,13]. Considering different culture and social environment between the western countries and China, our study tried to revise the contextual characteristic of the lasted Andersen model in Chinese socio-cultural context, and we selected predictors from it to study rural-to-urban migrant workers with NCMS. It would have some significance to the positive exploration of the Andersen Model (2013 Version) in the field of health in China.

Our study utilized a nationally representative dataset to determine the difference in health service utilization between rural migrant workers with NCMS in the county/district and rural migrant workers with NCMS across the county/district, and further decompose the differences in the health service utilization of two groups into its contributory factors at multiple levels of the lasted Andersen model. To enhance the comparability of the two groups, we used coarsened exact matching (CEM) to derive an estimator and control the confounding factors. Our findings may be referential for the off-site medical settlement in China for rural migrant workers, and the exploration of the policies of urban-rural resident basic medical insurance in China.

## 2. Methods

### 2.1. Data

The data sources of this study included two parts. At the micro level, the socio-economic data for our study were obtained from China Labor-Force Dynamic Survey in 2016 (CLDS 2016) which was carried out by the Center for Social Survey at Sun Yat-sen University. CLDS 2016 got the approval for the Biomedical Ethics Review Committee of Sun Yat-sen University (available online: http://css.sysu.edu.cn/Data (accessed on 30 August 2021)). In order to capture the differences in the public service capabilities of cities, our study used the community data of CLDS 2016, Urban Statistical Yearbook, and Statistical Bulletin to construct indicators at various levels. In order to reduce the possible endogenousness caused by reverse causality, we drew on the beneficial experience of previous research [14] and the data on urban service quality and other urban characteristics lagged one year. Therefore, the data of cities were obtained from the Urban Statistical Yearbook and Statistical Bulletin of the municipal government in 2015.

### 2.2. Study Population and Measurements

A total of 21,086 participants aged 15~64 participated in the survey of CLDS 2016. Our study focused on rural migrant workers participating in NCMS aged 15~64. The screening criteria for rural migrant workers participating in NCMS were as follows: aged 15–64, rural household registration, non-agricultural work, working hours of six months or more, and only participating in NCMS. 3322 respondents were included for further analysis.

In our study, the outcome variables were whether the rural migrant workers participating in NCMS visited a clinic or hospital services. Measurements of the health service utilization were based on the question: (1) Have you visited to the clinic at least one time within two weeks? (2) Have you been admitted to hospital during the past 12 months when the respondent was sick or injury? Two indicators were identified with dummy variables taking the value 1 if the respondent answered “yes”, and vice versa.

### 2.3. Predictor

Choosing critical predictors to explain the health service utilization patterns is very important. The lasted Andersen model emphasized the dynamics and circularity of four dimensions, but we only paid close attention to how different variables affect the health service utilization. Based on the availability of data and purposes of our study, the determinants of health service utilization are shown from the following aspects:

First, individual characteristics: age group (50~60; 61 and above), gender (male; female), living arrangement (live with spouse; live without spouse), educational level (below primary school; primary school; middle school and above), technical certificate (yes; no), type of industry (professional technician/clerical staff; service stuff; manufacturing and construction; freelancer), type of unit (party/government/state-owned/collective enterprises and institutions, private/foreign/joint venture, self-employed and freelance), working hours (moderate labor; excessive labor), income quantiles (poorest; poorer; middle; richer; richest), migration distance(in the county/district; across the county/district), injury insurance (yes; no), number of friends (<=5; 6~10; >=11), and self-assessed of health status (SAH) (good; fair; poor).

Second, health behavior: smoking (yes; no), alcohol use (yes; no), regular exercise every month (yes; no).

Third, health outcome: sense of fairness (unhappy; fair; happy).

Fourth, contextual characteristic: the proportion of ethnic minorities, per capita in the community, service quality index of the community, the service quality index of the city, health index of the community, region (east; central; west), city level reflecting the political rule and the policy-oriented factors in China (sub-provincial city and above; below sub-provincial city), the number of medical institutions per 10,000 people in the community, the number of medical institutions per 10,000 people in the city, the number of beds per 10,000 people in the city, and the number of doctors per 10,000 people in the city.

### 2.4. Coarsened Exact Matching

Migration out of the county/district presented a change of status for rural migrant workers with NCMS, and it was not randomly assigned [8]. NCMS is an endogenous variable for the health service utilization among rural migrant workers. There is inherent bias due to self-selection between rural migrant workers in the county/district and rural migrant workers across the county/district. A crude comparison of health service utilization between rural migrant workers in the county/district and rural migrant workers across the county/district would ignore the bias in the demographic composition of the two groups [15,16], so a straight comparison should be cautioned. Our study addressed the bias using CEM [17] in order to make two groups become (or very close to) identical in relation to individual characteristics. Compared to other matching methods, CEM can provide lower variance and bias for any sample size, and it does not require matched observations to be precisely similar in terms of these covariates [18,19,20]. Mark [21] proposed that if too many variables were included in the matching process, it would interfere with the matching process. He also proposed that robustness checks and robustness tests after CEM were not necessary [16,18]. Therefore, our study only selected variables that affect both health services utilization and work out of their own county/district. Age, sex, economic level and self-rated health were selected for matching, and the generated weights by CEM were used to equalize the number of two groups [16,18]. For balance checking before and after CEM, multivariate statistics is calculated based on the resulting distribution of matched cases and the selected covariates. L1 ranges from 0–1, with 0 indicts that bias has been removed, and 1 indicts a maximal imbalance. A lower L1 indicts a more balanced matching performance and thereby reducing the biases, and a weight can be used to determine the casual effect of the treatment effect [16,18]. “Cem” command code in Stata 15.0 [22] was developed by Blackwell, (not an official Stata command).

### 2.5. Multilevel Regression Model

The data showed a hierarchical structure of “city-community-rural migrant workers”. Rural migrant workers were nested in the community level unit, and then the community level was nested in the city unit. To capture within-group and between-group correlations in observations, a hierarchical linear regression model was employed. If only a conventional single-level regression model is used, we may violate the classic assumption of the single-level regression model-independent error terms and the “mean square error” of the city or community. Therefore, our study used multilevel regression models.

We used the Intra-Class Correlation Coefficient (ICC), that is, the ratio of the between-group variance to the total variance. The calculation formula of ICC is as follows:

(1) $$\mathrm{ICC}=\frac{\sigma_{u0}^{2}}{\sigma_{u0}^{2}+\sigma_{e0}^{2}}$$

$\sigma_{u0}^{2}$ presents the between-group variance, $\sigma_{e0}^{2}$ presents the within-group variance. ICC presents the degree of variation between groups. When the ICC is closer to 0, the individuals in the group tend to be independent, and the multilevel model can be simplified to a fixed-effect model. When the ICC is closer to 1, the difference between groups is larger than that within the group. When ICC is significantly larger than 0.059, multilevel regression models must be considered [23]. When the dependent variable is a binary variable, a linear approximation method in the generalized linear model needs to be used.

In general, basic operation steps of multilevel models can be divided into several steps. First, establish a null model, also known as an unconditional two-level model, to check the hierarchical structure of the data. ICC can be judged whether to use a multi-level model for analysis. Secondly, include variables representing the fixed effects to expand the null model to observe the significance of high-level explanatory variables. Thirdly, include the explanatory variable in level 1. The random slope of level 1 can be tested to adjust the effect of individual-level variables.

A three-level logistic regression model can be expressed as follows:

(2) $$\mathrm{logit}\left(\frac{p_{\mathrm{ijk}}}{1-p_{\mathrm{ijk}}}\right)={\beta x}_{\mathrm{ijk}}+{\gamma \omega }_{\mathrm{jk}}+{\eta z}_{k}+\mu_{\mathrm{jk}}+\nu_{k}$$

In the formula, i, j, k, represent level 3—city, level 2—community, and level 1—rural migrant workers with NCMS. Level 1—rural migrant workers with NCMS, level 2—community, and level 3—city, respectively, represent the estimated value of the regression coefficient of the explanatory variable at each level.

The three-level linear regression model can be expressed as follows:

(3) $$y_{\mathrm{ijk}}={\beta x}_{\mathrm{ijk}}+{\gamma \omega }_{\mathrm{jk}}+{\eta z}_{k}+\mu_{\mathrm{jk}}+\nu_{k}$$

$y_{\mathrm{ijk}}$ is a continuous dependent variable. i, j, k represent level 3 city, level 2 community, and level 1 rural migrant workers with NCMS. $x_{\mathrm{ijk}}$,$\omega_{\mathrm{jk}}$,$z_{k}$ represent the explanatory variables of level 1 rural migrant workers with NCMS, level 2 community, and level 3 city, respectively. β, γ, η represent the estimated value of the regression coefficient of the explanatory variable at each level. $\mu_{\mathrm{jk}},\nu_{k}$ represent the residuals of level 2 community and level 3 city, respectively.

### 2.6. Fairlie Decomposition

Since 0-non-use and 1-use in our study were binary, we utilized the decomposition technique proposed by Fairlie, which can decompose nonlinear models such as the logit, probit models [24,25,26,27]. Fairlie decomposition also allows identifying the observed differences between two groups differ from employment status. The influencing factors behind the difference between the two groups also can be showed as the Oaxaca-Blinder decomposition above. In line with the existing research [28], we randomly sorted the independent variables included in the model 100 times, the approximate average result represented the contribution of each explanatory variable to the difference. It is very difficult to explain the discrimination effect, so our study only focused the characteristic effect, not the discrimination effect, following the opinions of Fairlie [26].

The Fairlie decomposition can be written as follows [26,27]:

(4) $$\bar{Y_{u}}-\bar{Y_{r}}=\left[\sum_{i=1}^{N^{u}}\frac{F(X_{i}^{u}\beta^{u})}{N^{u}}-\sum_{i=1}^{N^{r}}\frac{F(X_{i}^{r}\beta^{u})}{N^{r}}\right]+\left[\sum_{i=1}^{N^{r}}\frac{F(X_{i}^{r}\beta^{u})}{N^{u}}-\sum_{i=1}^{N^{r}}\frac{F(X_{i}^{r}\beta^{r})}{N^{r}}\right]$$

$\bar{Y_{r}}$ and $\bar{Y_{u}}$ separately represent mean value of health service utilization of Chinese rural migrant workers with the New Cooperative Medical Scheme.r and u separately represented rural migrant workers in the county/district and rural migrant workers across the county/district.X represents independent variable, β represents the coefficient.$N^{r}$ and $N^{u}$ separately represent sample sizes of rural migrant workers in the county/district and rural migrant workers across the county/district. The first bracket represents the characteristic. The second bracket represents the discrimination effect, that is the differences caused by different characteristic regression coefficients [28]. A positive coefficient represents a positive contribution to the difference, and vice versa. The sample needs to be weighted, and the decomposition command can be slightly modified in the operating software.

## 3. Result

### 3.1. Matching Performance

Match balance test is required before and after matching. Table 1 showed the multivariate statistics before and after matching. After CEM, between rural migrant workers in the county/district and rural migrant workers across the county/district was actually close to zero (9.07 × 10^−16^), which were much lower than that before matching (0.252), revealing a good matching performance.

After CEM, the sample size of rural migrant workers with NCMS in this county/district may not be equal to the size of rural migrant workers across the county/district, so weights would be assigned in the CEM process. Table 2 showed that it was obvious that there was no statistically significant difference on all the matching characteristic between rural migrant workers with NCMS in the county/district and rural migrant workers with NCMS across the county/district after matching, which also indicated a good matching performance and thus the two groups became more comparable. After matching, there were 2280 rural migrant workers with NCMS in the county/district and 427 rural migrant workers with NCMS across the county/district.

### 3.2. Logit Regression Analysis

Table 3 presented the estimations of three-level (personal level, community level, and city level) regression models. The variance of city level and community level of the two-week outpatient probability of rural migrant workers with NCMS in the county/district were 0.396 and 0.109, respectively. Then, the ICC of city level and community level can be calculated to be 0.104 and 0.133, respectively. The variance of city level and community level of the inpatient probability of rural migrant workers with NCMS in the county/district were 2.81 × 10^−35^ and 3.41 × 10^−34^, respectively. Then, the ICC of city level and community level can be calculated to be 8.53 × 10^−36^ and 1.12 × 10^−34^, respectively. The variance of city level and community level of the inpatient probability of rural migrant workers with NCMS across the county/district was 5.01 × 10^−33^ and 0.002, respectively. The variance of city level and community level of inpatient probability were 0.294 and 5.01 × 10^−33^, respectively. The ICC of inpatient probability at city level and community level were 0.082 and 0.082, respectively. Therefore, it is necessary to use multilevel regression models to analyze the two-week outpatient probability of rural migrant workers with NCMS in the county/district, the two-week outpatient probability of rural migrant workers with NCMS across the county/district and inpatient probability of rural migrant workers with NCMS across the county/district.

Table 4 presented the association of independent variables and health service utilization by multilevel regression models. The factors influencing the two-week outpatient utilization for rural migrant workers with NCMS in the county/district were marital status, injury insurance, number of friends, SAH and regular exercise every month; the factors influencing the inpatient utilization for rural migrant workers with NCMS in the county/district was SAH; the factors influencing the two-week outpatient utilization for rural migrant workers with NCMS across the county/district were occupational type, economic level, SAH and regular exercise per month; the factors influencing the inpatient utilization for rural migrant workers with NCMS across the county/district were gender, occupation, type of organization, exercise, and well-being.

### 3.3. Fairlie’s Decomposition of Differences in Health Service Utilization

Table 5 showed the contribution of characteristics to the explained difference in the probability of health service utilization using regression-based decomposition results. The difference of two-week outpatient service utilization between rural migrant workers with NCMS in the county/district and rural migrant workers with NCMS across the county/district was −0.004, and the difference of inpatient service utilization was −0.008. That is, the rural migrant workers with NCMS in the county/district reported a higher health service utilization than rural migrant workers with NCMS across the county/district. Our results explored to what extent the differences are due to population characteristics rather than to unexplained factors associated with migration status. About 23.10% of the total difference of two-week outpatient service utilization was due to the observed influence factors. About 87.22% of the total difference of inpatient service utilization was due to the observed influence factors. Table 5 showed the proportion of ethnic minorities, the number of medical institutions for 10,000 people in the community, the number of beds for 10,000 people in the city, the urban service quality index were the major contributors. Table 5 also showed that the proportion of difference in the health service utilization of rural migrant workers with NCMS caused by health service need were −54.73% and 6.92%, respectively. The inequities of the probability of two-week outpatient service and the probability of inpatient service were −0.006 and −0.007, respectively.

## 4. Discussion

Although NCMS has provided benefits for the health service utilization of rural migrant workers, there is scare study on the health services utilization in light of the strong regional segmentation of NCMS. To the best of our knowledge, this study is the first comparative study to pay the attention to the health service utilization difference and equity of rural migrant workers from the perspective of off-site medical treatment of NCMS. Our study documented that the newly added categories to the Andersen Model (2013 Version) in Chinese context, and we applied it to explore factors on health service utilization, which can expand the rational thinking on the health service utilization of rural migrant workers with NCMS.

Comparing rural migrant workers with NCMS across the county/district whose occupation is service personnel, production and related personnel, and self-employed, the outpatient probability of those whose occupation is professional technical/clerical personnel is significantly higher. It is probably since their work is relatively stable and their income is relatively stable. The results also reminded that the employer should give rural migrant workers sick leave to avoid delay in the timely treatment of disease. Otherwise, it may lead rural migrant workers to further avoid seeking healthcare in the early stages of disease if the healthcare seeking process becomes more arduous or time-consuming. In lines with previous studies [29], social support can improve physical health. Rural migrant workers with NCMS in the county/district with more than 11 friends were less likely to see doctors for two weeks, consistent with other studies [30,31] that friends can promote a healthy life.

Rural migrant workers with NCMS across the county/district were at a disadvantage in the health service utilization. On one hand, it can be explained by the effect of health selection (that is, rural migrant workers with better health status tend to migrate over a greater distance). On the other hand, although the policy of off-site medical treatment of NCMS has made remarkable progress in China, the problem of off-site medical treatment of NCMS is still very prominent. Considering different policies on NCMS implemented by different countries/districts, rural migrant workers with NCMS may be less well informed about the policies on NCMS. Thus, it is urgent to improve the information management systems and off-site medical treatment system, to ensure the transfer and continuity of health insurance of different places. Compared with differences in the probability of two-week outpatient service utilization, the discriminatory effect had a smaller impact on the difference in the probability of inpatient service utilization. Our study showed that smoking had a significant impact on the difference in two-week outpatient service utilization of two matched groups, illustrating the importance of healthy lifestyle and tobacco control. The opposite of happiness is relative deprivation. Rural migrant workers tend to pay more attention to the people and things around them; rural migrant workers would like to compare the reference object. In fact, social comparison occurred mainly within homogeneous groups in western developed countries [32,33].

There are substantial differences in financial health expenditure and compensation for NCMS among different countries/districts, which increased the difference in the health service utilization. Both the number of medical institutions for 10,000 population and service quality index of the city played important roles in explaining the overall gap in two-week outpatient service utilization between the two matched groups. Therefore, we should pay attention to improving the quality of public service in communities and cities where rural migrant workers lived in. The higher number of medical institutions for 10,000 population in communities, beds for 10,000 population in cities and urban service quality index meant a better access to health services in cities and communities. Therefore, the difference of inpatient probability between rural migrant workers with NCMS in the county/district and rural migrant workers with NCMS across the county/district enlarged.

It is suggestive to adjust and perfect the schemes further. Due to the particularity of the work characteristics and social status of rural migrant workers with NCMS, their gender, age, and health status may affect their health service needs. Excessive pursuit of equality in their health service utilization without paying attention to their health service needs may lead to more serious inequality. Therefore, we should accurately find out the health service need of rural migrant workers with NCMS on the basis of full investigation. What’s more, the implementation of basic health insurance for rural migrant workers has put forward higher top-level design and higher requirements for Chinese government. The more practical way is to realize the unified integration and transfer of the medical insurance relationship of rural migrant workers between different basic medical insurance systems. It is also important to note that the real-time settlement of medical treatment in different places needs to realize the information management of medical insurance and the efficient cooperation of designated medical institutions. The medical insurance information of rural migrant workers with NCMS can be managed with the help of cutting-edge technologies such as internet technology and artificial intelligence.

There is some caution, however. We would also like to note several limitations in our study. First, our study described a cross-sectional analysis that cannot allow the determination of time precedence or causal inferences between health service utilization and related factors, and in future we will strive to obtain data at different times and preform a longitudinal tracking study. Second, considering the availability of data, our study was limited to the use of health services and health outcomes for many variables in the lasted Andersen model. Future work could expand this effort by understanding other conditions and exploring in detail the reasons for the health services use of rural migrant workers. Third, the limited sample size for the rural migrant workers included also did not allow a detail analysis with any given condition and with each of individual characteristics, health behavior, health outcome, and contextual characteristic. Future studies with a sufficiently large sample sizes should be conducted to ensure adequate power.

## 5. Conclusions

Our study highlighted substantial differences in the health service utilization between rural migrant workers with NCMS in the county/district and rural migrant workers with NCMS across the county/district. The factors influencing health service utilization were diversified. Based on our findings, it is clear that health service needs of the rural migrant workers with NCMS should be fully considered, and we clarified the inequity on the basis of the characteristic effect and the discrimination effect, which enriched the explanation of the differences in health service utilization inequality of rural migrant workers with NCMS. Our study may help to offer evidence for future social policy and intervention strategies targeted to the construction of health insurance system with pertinence, focusing on health service utilization.

## Figures and Tables

**Table 1:** The L_1_ measure of imbalance before and after CEM.

Variables	Before Matching		After Matching	
	L_1_	Mean	L_1_	Mean
Age	0.099	0.185	9.30 × 10^−16^	6.00 × 10^−15^
Gender	0.051	−0.050	6.70 × 10^−16^	2.40 × 10^−15^
Quintiles	0.16	−0.452	1.10 × 10^−15^	5.80 × 10^−15^
SAH	0.015	0.028	4.90 × 10^−16^	1.60 × 10^−15^
Multivariate L_1_	0.252		9.07 × 10^−16^	

**Table 2:** Summary statistics for key variables after coarsened exact matching.

Variables	Before Matching N (%)			After Matching N (%)		
	In the County/District	Across the County/District	*p*	In the County/District	Across the County/District	*p*#
Age group			<0.001			0.781
15~36 †	1018 (37.41)	285 (47.42)		892 (39.12)	165 (38.55)	
36~50	972 (35.72)	227 (37.77)		867 (38.03)	162 (38.05)	
50~64	731 (26.87)	89 (14.81)		521 (22.85)	100 (23.40)	
Gender			<0.01			0.973
Men †	1593 (58.54)	317 (52.75)		1340 (58.77)	251 (58.86)	
Women	1128 (41.460)	284 (47.25)		940 (41.23)	176 (41.14)	
Quintiles			<0.001			0.842
Poorest †	506 (20.64)	69 (16.24)		470 (20.61)	93 (21.88)	
Poorer	526 (21.46)	49 (11.53)		486 (21.32)	76 (17.77)	
Middle	504 (20.56)	72 (16.94)		466 (20.44)	93 (21.82)	
Richer	462 (18.85)	113 (26.59)		432 (18.95)	87 (20.29)	
Richest	453 (18.48)	122 (28.71)		426 (18.68)	78 (18.24)	
SAH			0.091			0.860
Good †	1864 (68.50)	421 (70.05)		1603 (70.31)	296 (69.32)	
Fair	685 (25.17)	152 (25.29)		577 (25.31)	111 (26.00)	
Poor	172 (6.32)	28 (4.66)		100 (4.39)	20 (4.68)	
N	2721	601		2280	427	

Note: † Reference levels in the regressions; virtual variables for Chi-square test; *p*-value indicated the actual *p*-values after matching; *p*#-value indicated the weight to be considered; N (%) were reported. In the county/district presented rural migrant workers with NCMS in the county/district. Across the county/district presented rural migrant workers with NCMS across the county/district.

**Table 3:** Three-level empty model of influencing factors of health service utilization between the matched groups.

Variable		Outpatient/In		Inpatient/In		Outpatient/Across		Inpatient/Across	
		OR	SE	OR	SE	OR	SE	OR	SE
Fixed effects	Intercept	−3.042 ***	0.149	−2.773 ***	0.083	−2.982 ***	0.268	3.205 ***	0.390
Random effects	City level variance	0.396	0.231	2.81 × 10^−35^	2.93 × 10^−35^	5.01 × 10^−33^	5.29 × 10^−32^	0.294	0.438
	Community level variance	0.109	0.199	3.41 × 10^−34^	7.94 × 10^−15^	0.012	0.547	1.21 × 10^−33^	1.63 × 10^−33^
	Personal level parameter	1.000	0.000	1.000	0.000	1.000	0.000		

Note: Estimates of random-effect parameters and residual variance parameters were reported as standard errors. SE for standard error. Outpatient/In presented the two-week outpatient probability of rural migrant workers with NCMS in the county/district; Inpatient/In presented the inpatient probability of rural migrant workers with NCMS in the county/district; Outpatient/Across presented the two-week outpatient probability of rural migrant workers with NCMS across the county/district; Inpatient/Across presented the inpatient probability of rural migrant workers with NCMS across the county/district; *** *p* < 0.001.

**Table 4:** Association of independent variables and health service utilization.

Variables	Outpatient/In		Inpatient/In		Outpatient/Across		Inpatient/Across	
	OR	SE	OR	SE	OR	SE	OR	SE
Individual characteristics								
Age group								
15~36 †								
36~50	0.871	0.218	0.800	0.202	0.528	0.394	0.194	0.240
50~64	0.716	0.226	1.278	0.348	0.876	0.763	0.508	0.283
Gender								
Men †								
Women	0.848	0.227	1.426	0.393	4.161	4.009	19.984 **	6.597
Living arrangement								
Live with spouse †								
Live without spouse	0.537 *	0.145	1.401	0.440	1.773	1.636	1.142	0.998
Educational attainment								
Below primary school †								
Primary school	1.036	0.262	1.388	0.332	3.809	3.186	0.308	0.264
Middle school and above	1.028	0.315	1.162	0.360	1.495	1.779	0.251	0.243
Technical certificate								
Yes †								
No	0.960	0.324	0.617	0.172	1.798	2.080	0.127 *	0.107
Type of industry								
Professional technician/Clerical staff †								
Service stuff	0.766	0.358	1.339	0.636	0.079 *	0.101	15.338 **	13.819
Manufacturing and construction	0.708	0.334	1.843	0.892	0.028 *	0.041	1.411	2.257
Freelancer	0.721	0.408	1.644	0.870	0.005 ***	0.008	22.061	36.979
Type of unit								
Party/government/state-owned †								
Collective enterprises and institutions	1.010	0.384	1.105	0.422	1.105	0.422	10.812 **	7.561
Self-employed and freelance	1.067	0.464	1.017	0.401	1.017	0.401	1.379	0.401
Working hours								
Moderate labor †								
Excessive labor	1.135	0.245	0.926	0.180	1.996	1.295	2.128	1.253
Income quintiles								
Poorest †								
Poorer	0.975	0.230	1.048	0.314	0.117	0.145	0.543	0.460
Middle	0.719	0.186	1.021	0.306	0.054 *	0.069	0.893	0.440
Richer	1.117	0.316	0.974	0.320	0.402	0.298	0.198	0.174
Richest	0.474	0.182	1.584	0.514	0.289	0.269	0.347	0.289
Injury insurance								
Yes †								
No	0.543 *	0.169	0.819	0.290	0.803	0.632	2.532	3.517
number of friends								
<= 5 †								
6~10	1.038	0.220	0.875	0.201	1.260	0.835	0.230	0.263
>=11	0.504 *	0.170	1.024	0.258	0.353	0.353	0.700	0.634
SAH								
Good †								
Fair	3.448 ***	0.889	−0.051	0.250	5.663 *	3.981	1.309	0.622
Poor	8.715 ***	3.182	0.427	0.302	17.599 ***	7.694	0.444	1.243
Health behavior								
Smoke								
Yes †								
No	1.312	0.426	−0.332	0.331	0.129	0.134	0.033	0.035
Alcohol use								
Yes †								
No	1.221	0.392	−0.363	0.292	1.927	1.563	0.163	0.231
Regular exercise every month								
Yes †								
No	0.656 *	0.123	−0.109	0.252	11.919	12.816	0.158 ***	0.084
Health outcome								
Sense of happiness								
Unhappy †								
Fair	0.999	0.386	0.546	0.515	0.311	0.302	0.112	0.134
Happy	0.784	0.300	0.488	0.479	0.525	0.478	0.133	0.178
Contextual characteristic								
Proportion of ethnic minorities	1.000	0.006	0.175 *	0.006	0.990	0.070	0.965	0.083
Per capita in the community	1.000	2.02 × 10^−4^	1.64 × 10^−5^	1.15 × 10^−5^	1.000	0.000	1.000	0.000
Region								
East †								
Middle	0.890	0.285	−0.192	0.300	0.856	1.007	4.840	5.053
West	0.872	0.352	−0.042	0.349	0.112	0.161	0.403	0.615
City level								
Sub-provincial city and above								
Other	1.261	0.428	0.068	−0.294	0.538	0.753	0.27	0.103
Number of medical institutions for 10,000 people in the community	3.96 × 10^−5^	4.14 × 10^−4^	−1.135	−0.388	7.22 × 10^−12^	2.74 × 10^−25^	0.858	0.579
Number of medical institutions for 10,000 people in the city	1.003	0.102	−0.277 *	−0.117	0.250	0.767	0.979	0.016
Number of doctors for 10,000 people in the city	1.002	0.004	0.001	−0.003	1.014	0.013	1.020	0.014
Number of beds for 10,000 people in the city	0.985	0.011	−0.006	−0.01	0.986	0.013	0.654	0.645
Health index of the community	0.872	0.129	0.401	−0.317	0.829	0.948	1.403	0.844
Service quality index of the community	1.086	0.174	0.049	−0.133	0.850	0.314	0.425	0.358
Urban service quality index	0.985	0.226	0.174	−0.221	0.170	0.174	0.364	0.358
Intercept	0.296	0.277	10.360 ***	−1.02	0.311	0.302	0.112	0.134

Note: † Reference levels in the regressions; SE, standard error. Outpatient/In presented the two-week outpatient probability of rural migrant workers with NCMS in the county/district; Inpatient/In presented the inpatient probability of rural migrant workers with NCMS in the county/district; Outpatient/Across presented the two-week outpatient probability of rural migrant workers with NCMS across the county/district; Inpatient/Across presented the inpatient probability of rural migrant workers with NCMS across the county/district; * *p* < 0.05, ** *p* < 0.01, *** *p* < 0.001.

**Table 5:** Fairlie’s decomposition of the difference of health service utilization between the matched groups.

Terms of Decomposition	Two-Week Outpatient Service		Inpatient Service	
Total gap (%)	−0.004			−0.008
Explained (%)	23.10			87.22
Explained				
Contribution to difference	Coef.	Contribution (%)	Coef.	Contribution (%)
Age group	−0.007	1.57	−0.130	10.53
Gender	0.051	−11.93	0.046	−3.76
Living arrangement	0.027	−6.24	0.009	−0.76
Educational level	2.99 × 10^−4^	−0.002	−0.116	9.41
Technical certificate	−0.037	8.56	−0.170	13.81
Type of industry	0.506	−117.61	0.058	−4.69
Type of unit	0.037	−8.56	0.236	−19.14
Working hours	−0.023	5.41	0.063	−5.12
Income quantiles	0.046	−10.63	0.048	−3.85
Injury insurance	−0.005	1.19	0.019	−1.5
Number of friends	0.126	−29.36	0.449	−36.35
SAH	0.191	−44.37	−0.002	0.15
Smoking	−0.353	82.17	−0.207	16.73
Alcohol use	−0.025	5.74	0.070	−5.65
Regular exercise every month	0.073	−16.88	−0.323	26.15
Sense of fairness	−0.096	22.39	0.001	−0.05
Proportion of ethnic minorities	0.176	−40.86	−0.162	13.15
Per capita in the community	−0.095	22.01	0.067	−5.41
Region	−0.251	58.29	0.596	−48.27
City level	0.045	−10.52	−0.039	3.15
Number of medical institutions for 10,000 people in the community	0.175	−40.73	−1.744	141.29
Number of medical institutions for 10,000 people in the city	−0.096	22.36	−0.064	5.15
Number of doctors for 10,000 people in the city	−0.386	89.74	2.265	−183.44
Number of beds for 10,000 people in the city	0.369	−85.93	−1.461	118.35
Health index of the community population	−0.516	119.97	−1.375	111.36
Service quality index of community	−0.080	18.64	0.121	−9.84
Service quality index of city	0.049	−11.35	0.669	−54.18
Difference caused by need	0.002	−54.73	−5.54 × 10^−4^	6.92
Inequity index	−0.006	154.73	−0.007	93.08

Note: A logit regression model on a pooled sample was run; Coef., Coefficient; SE, standard error.

## Data Availability

All the data we used have been publicly released on the CLDS website: http://css.sysu.edu.cn/Data. (accessed on 30 August 2021).

## Abbreviations

CLDS	China Labor-Force Dynamic Survey
CEM	coarsened exact matching method
Hukou	Chinese household registration system
NCMS	New cooperative medical scheme
95% CI	95% Coefficient Interval
